# Efficacy of Extracorporeal Shock Wave Therapy for the Treatment of Chronic Pelvic Pain Syndrome: A Randomized, Controlled Trial

**DOI:** 10.1155/2013/972601

**Published:** 2013-08-28

**Authors:** Babak Vahdatpour, Farshid Alizadeh, Amir Moayednia, Masoud Emadi, Mohammad Hatef Khorami, Saeid Haghdani

**Affiliations:** ^1^Department of Physical Medicine and Rehabilitation, Isfahan University of Medical Sciences, Isfahan, Iran; ^2^Department of Urology, Isfahan Urology and Kidney Transplantation Research Center, Isfahan University of Medical Sciences, Unit 10, No. 22, 16th Alley, Shams Abadi Street, Isfahan 81347-44134, Iran; ^3^Department of Urology, Hasheminejad Kidney Center (HKC), Tehran University of Medical Sciences (TUMS), Tehran, Iran

## Abstract

*Objectives*. To investigate the effectiveness of extracorporeal shock wave therapy (ESWT) for symptoms alleviation in chronic pelvic pain syndrome (CPPS). *Materials and Methods*. 40 patients with CPPS were randomly allocated into either the treatment or sham group. In the first group, patients were treated by ESWT once a week for 4 weeks by a defined protocol. In the sham group, the same protocol was applied but with the probe being turned off. The follow-up assessments were done at 1, 2, 3, and 12 weeks by Visual Analogue Scale (VAS) for pain and NIH-developed Chronic Prostatitis Symptom Index (NIH-CPSI). *Results*. Pain domain scores at follow-up points in both treatment and sham groups were reduced, more so in the treatment group, which were significant at weeks 2, 3, and 12. Urinary scores became significantly different at weeks 3 and 12. Also, quality of life (QOL) and total NIH-CPSI scores at all four follow-up time points reduced more significantly in the treatment group as compared to the sham group. Noticeably, at week 12 a slight deterioration in all variables was observed compared to the first 3 weeks of the treatment period. *Conclusions*. our findings confirmed ESWT therapy as a safe and effective method in CPPS in short term.

## 1. Introduction

Chronic pelvic pain syndrome (CPPS) is a frequent outpatient urological diagnosis [1]. The incidence is increasing, being reported to be around 15% [2–4]. Symptoms of CPPS are urinary and erectile dysfunctions and pain in the prostate, perineal, inguinal, scrotal, and suprapubic regions, lasting for at least 3 of the previous 6 months [5–7]. The quality of life (QOL) is also disturbed as the result of urinary and erectile dysfunctions [8, 9]. Greater pain and urinary symptoms are associated with worse QOL [10, 11].

The pathophysiology of CPPS has not yet been completely explained. Psychiatric and somatic factors possibly play roles; however, no infection or bacterial pathogen has been detected yet [12]. Moreover, myofascial pain syndrome along with a neurological component has been associated with dysfunctional effects of this disease [13–15]. Medical therapies such as analgesics, anti-inflammatory agents, antibiotics, *α*-receptor blockers, and 5*α*-reductase inhibitors have been used as single or combination therapy with variable success rates [7, 16, 17]. 

There are also some alternative therapies that have been introduced such as physiotherapy, trigger-point massage, electromagnetic treatment, acupuncture, rectal massage, hyperthermia, thermotherapy, balloon dilatation, laser coagulation, invasive neuromodulation, and intraprostatic injection of botulinum toxin A [18, 19]. None of these modalities, however, has been uniformly successful.

Recently, effectiveness of perineal extracorporeal shock wave therapy (ESWT) has been investigated in CPPS patients [3, 6, 20, 21]. There are different mechanisms through which ESWT reduces pain: interrupting the flow of nerve impulses by hyperstimulation of nociceptors, healing tissue by revascularization processes, and reductions in muscle tone and spasticity [3, 20, 21].

In this study we conducted a randomized sham-controlled trial to evaluate the efficacy of ESWT on CPPS.

## 2. Materials and Methods 

### 2.1. Participants

From October 2011 to October 2012, all patients with chronic prostatitis type IIIB/chronic pelvic pain syndrome according to NIH International Prostatitis Collaboration Network reports [22] who were referred to Urology Clinic of Al-Zahra Hospital were enrolled in this study. Based on the actual National Institutes of Health (NIH) classification [23], CPPS (type IIIB) is characterised by the lack of signs of infection in urine and sperm as well as by the specific symptoms. Eligible patients signed an informed consent. The study inclusion criteria were as follows: nonaddiction to drugs and narcotics, chronic pelvic pain existence for more than three months, and certain diagnosis of chronic nonbacterial/chronic pelvic pain syndrome defined as pain in the bladder, groin, genitalia or lower abdomen, and/or perineal areas without clear abnormalities on urological examination. A renal and bladder ultrasound was done in all patients to check for bladder or lower ureteral stones and whenever indicated, a retrograde urethrography was requested for ruling out any urethral pathologies. The exclusion criteria of this study included being under treatment by another method at the beginning of the study, another diagnosis such as prostate cancer during workup, therapy plan alteration, and noninclination to continue this project.

Bacterial prostatitis was ruled out by a 2-glass test. In this test a midstream urine sample was collected with the 10 CC of urine being discarded and the second 10 CC collected and then prostate massage was done for a minute by digital rectal exam and then another 10 cc of urine was collected. These samples were analysed and cultured.

### 2.2. Method of Treatment

The study protocol was approved by the Ethics Committee of Isfahan University of Medical Sciences. After patient consultation about the method and obtaining written consent, they were allocated into either the treatment or sham group with simple randomization. In the first group, patients were treated by ESWT once a week for 4 weeks. Each time 3000 impulses, with 0.25 mJouls/mm^2^ and 3 Hertz of frequency were delivered, although 0.5 mJouls/mm^2^ was added in each week (0.3 mJouls/mm^2^ in week two, 0.35 mJouls/mm^2^ in week three, and 0.4 mJouls/mm^2^ in week four). After each 500 pulses, the probe position was corrected, using transperineal ultrasound. In this study we used the standard electromagnetic DUOLITH SD1, Storz Medical, Tägerwilen, Switzerland. The treatment was performed in supine position.

In the sham group, the same protocol was applied but with the probe being turned off.

### 2.3. Evaluation of Results

The follow-up assessments were done at 1, 2, 3, and 12 weeks following the first ESWT session. For each patient, pelvic pain intensity was measured at the beginning of each follow-up episode, using Visual Analogue Scale (VAS, 0–10). In addition, NIH-developed Chronic Prostatitis Symptom Index (NIH-CPSI) was filled at the beginning of each follow-up visit. Finally, obtained data were recorded in special profile for each patient and analysed.

### 2.4. Statistical Analysis

Data were entered in SPSS (version 18). The statistical analyses such as chi-square, independent *t*-test, Wilcoxon, and, as necessary, repeated ANOVA were used.

## 3. Result

The mean ages of the patients in the treatment and sham groups were 35.4 ± 8.4 and 37 ± 10.1 years, respectively. At baseline, the means of pain score, urinary score, QOL, and NIH-CPSI score between the two groups were not statistically different (Table 1). Pain domain scores were reduced in both treatment and sham groups, although the difference became statistically significant after the second treatment session. Urinary score was significantly different between the two groups only at weeks 3 and 12. In addition, QOL and total NIH-CPSI scores at all four follow-up time points in the treatment group decreased more significantly as compared to the sham group.

It should be noticed that in all four domains, as well as the pain score, some deterioration was observed at week 12 compared to week 3 of followup (Table 1). Repeated measurements of ANOVA findings revealed that pain domain, urinary score, QOL, and total NIH-CPSI score in the treatment group during the 3-week treatment period were improved; however at week 12 of followup, a slight deterioration in symptoms was observed. On the other hand, in the sham group a mild decrease was observed in all variables during the 3-week treatment period; however, at week 12 values increased and returned to the baseline, whereas in the treatment group the values were still significantly lower than in the baseline.

None of the patients experienced perineal pain or voiding difficulty during the follow-up period.

**Figure 1:**
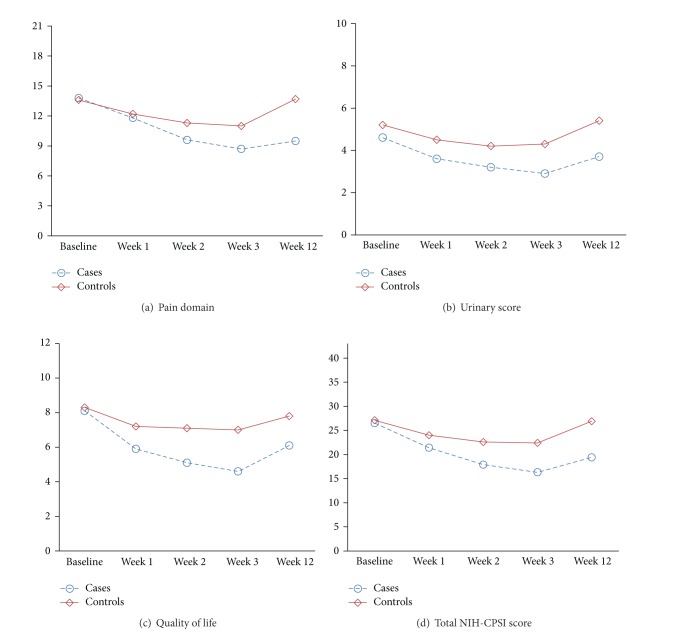
Comparison of trend of variables changes between study groups. (a) Pain domain, (b) urinary score, (c) quality of life, and (d) total NIH-CPSI score. Differences in the scores of pain domain (*P* value = 0.001), urinary score (*P*-value = 0.039), quality of life (*P*-value < 0.0001), and total NIH-CPSI score (*P*-value < 0.0001), between cases and controls were statistically significant. *x*-axis: time points. *y*-axis: mean of scores. *P*-values calculated by repeated measurements of ANOVA.

Totally, results in Figure 1 showed that improvements in the values of pain domain, urinary score, QOL, and total NIH-CPSI scores during study period in treatment group were significantly better than those in sham group.

## 4. Discussion

Our study showed that total NIH-CPSI, pain and urinary symptom scores, and QOL improved significantly in ESWT group compared to sham group, although we observed some deterioration in all fields at week 12 of follow up compared with week 3.

In recent years, a few studies have evaluated the efficacy of ESWT on CPPS. Zimmermann et al. in their first study [6] showed statistically significant improvements in pain and quality of life after ESWT. Voiding conditions as measured by International Prostate Symptom Score (IPSS) improved but with no statistical significance. In their cohort, only 17% of patients showed an increase in serum PSA two days after treatment and in the others, the increase was less than 10% or even a reduction was observed. This finding shows that ESWT is not traumatic for the prostate gland. In our patients, no pain or discomfort was observed during or after treatment.

Later, Zimmermann et al. reported a similar trial [20] that included 60 patients in which they used National Institutes of Health Chronic Prostatitis Symptom Index (NIH-CPSI), International Prostate Symptom Score (IPSS), International Index of Erectile Function (IIEF), and the Visual Analog Scale (VAS) to investigate their parameters. They found reduced pain and improved QOL in a significantly greater proportion of patients who underwent ESWT treatment. Interestingly, patients in the control group showed no improvement despite what one would expect from the placebo effect. In our study, an improvement in symptoms was observed in both treatment and sham groups. However, the difference became significant at the second week for the pain score and at the third week for the urinary score and remained so until the 12 week of followup (Table 1). Although some deterioration in symptoms occurred at week 12 in the treatment group, NIH-CPSI was still significantly less than in the baseline. In the control group, however, symptom score returned to the baseline after 12 weeks. 

Yan et al. [21] in their randomized study with 80 CPPS patients revealed that NIH-CPSI, quality of life (QOL), and the pain domain scores significantly improved compared to the baseline at all posttreatment time points in ESWT group. At the end-point of treatment, 71.1% of ESWT group exhibited perceptible improvement in total NIH-CPSI compared with 27.0% of sham group; moreover, 28.9% of the ESWT group exhibited clinically significant improvement compared with 10.8% of the sham group.

One important issue is the exacerbation of NIH-CPSI, pain, and symptom scores on week 12 followup in both groups that may challenge the persistence of therapeutic effect of ESWT therapy. In previous surveys the outcomes showed only progress in their trend on followup, which is not in accordance with our results. Therefore, more comprehensive research with long-term followup may clarify this ambiguous point.

The pathogenesis of the CPPS is not completely understood. Proposed mechanisms include infection leading to pain via nociceptive nerve endings and receptors, pelvic floor hyperactivity, local chemical alterations, neurologic components, and perfusion disturbances [3, 12, 13]. The role of the prostate is challenging [13]. Extracorporeal shock waves affect the tissue by transformation of mechanical signals into biochemical or molecular biologic signals [20]. There are some explanations that ESWT modulates the transmission of pain signal, such as producing extracellular cavitation when passing through human tissues that may result in damage to local nerve endings, activating the small-diameter fibres and the serotonergic system and finally, the gate-control theory [24–26]. All over, although no consensus exists about the mechanism of ESWT on CPPS, reducing passive muscle tone, hyperstimulating nociceptors, interrupting the flow of nerve impulses, and influencing the neuroplasticity of the pain memory are some considerations [6, 27–29].

Numerous studies in orthopaedics, urology, and cardiology have shown ESWT to have low side effects [6, 26, 27, 29]. Lack of PSA rise in Zimmermann's study confirms this fact. 

ESWT effect can be considered dose dependent [20, 30]. In our study, the numbers of shock waves and the energy level were empirical. The selection of the number of treatments, the treatment intervals, and the number of pulses per session was made according to clinical studies of previous applications. In our study protocol, a modification was made and 0.5 mJoule/mm^2^ was added in each week. Patients showed improvement in their symptoms in week 3 compared to week 2; however this improvement did not continue until week 12 and consequently no definitive conclusion could be drawn regarding the long-term effect of this study protocol. One of the shortcomings of our study is that we did not evaluate the IPSS score and erectile function in our patients. The effectiveness of different treatment intervals and frequencies must be investigated further to define optimum treatment regimens for ESWT effects.

## 5. Conclusion 

In conclusion, our findings confirmed ESWT to be a safe and effective therapy for CPPS in the short term. Nevertheless, more comprehensive surveys so as to describe a standard protocol for ESWT, with long-term followups, are essential.

## Figures and Tables

**Table 1:** Comparison of the mean of pain domain, urinary score, QOL, and NIH-CPSI scores between study groups at time points.

	Time point				
	Baseline	Week 1	Week 2	Week 3	Week 12
Pain domain					
Case	13.8 ± 2.6	11.8 ± 2.2	9.6 ± 1.7	8.7 ± 1.5	9.5 ± 0.9
Control	13.6 ± 2	12.2 ± 1.7	11.3 ± 1	11 ± 0.7	13.7 ± 1.6
*P* value	0.78	0.53	0.001	<0.0001	<0.0001
Urinary score					
Case	4.6 ± 2.8	3.6 ± 2.2	3.2 ± 1.9	2.9 ± 1.5	3.7 ± 1.5
Control	5.2 ± 2	4.5 ± 1.8	4.2 ± 1.4	4.3 ± 0.9	5.4 ± 1.3
*P* value	0.44	0.19	0.051	0.001	0.001
Quality of life					
Case	8.1 ± 1.7	5.9 ± 1.5	5.1 ± 1.5	4.6 ± 1.3	6.1 ± 0.8
Control	8.3 ± 1.9	7.2 ± 1.6	7.1 ± 1.2	7 ± 0.7	7.8 ± 0.9
*P* value	0.73	0.01	<0.0001	<0.0001	<0.0001
NIH-CPSI score					
Case	26.5 ± 3.4	21.4 ± 2.7	17.9 ± 2.5	16.3 ± 2.1	19.4 ± 1.4
Control	27.1 ± 3.1	24 ± 2.8	22.6 ± 2.2	22.4 ± 1.1	26.9 ± 3
*P* value	0.56	0.006	<0.0001	<0.0001	<0.0001

Data are mean ± SD.

*P* values calculated by independent samples test.
